# Big knowledge from big data in functional genomics

**DOI:** 10.1042/ETLS20170129

**Published:** 2017-11-14

**Authors:** Chris P. Ponting

**Affiliations:** MRC Human Genetics Unit, The Institute of Genetics and Molecular Medicine, University of Edinburgh, Western General Hospital, Crewe Road, Edinburgh EH4 2XU, U.K.

**Keywords:** genome annotation, genome editing, Mendelian randomisation, reverse genetics

## Abstract

With so much genomics data being produced, it might be wise to pause and consider what purpose this data can or should serve. Some improve annotations, others predict molecular interactions, but few add directly to existing knowledge. This is because sequence annotations do not always implicate function, and molecular interactions are often irrelevant to a cell's or organism's survival or propagation. Merely correlative relationships found in big data fail to provide answers to the *Why* questions of human biology. Instead, those answers are expected from methods that causally link DNA changes to downstream effects without being confounded by reverse causation. These approaches require the controlled measurement of the consequences of DNA variants, for example, either those introduced in single cells using CRISPR/Cas9 genome editing or that are already present across the human population. Inferred causal relationships between genetic variation and cellular phenotypes or disease show promise to rapidly grow and underpin our knowledge base.

Single-gene studies in model or cellular systems have substantially advanced knowledge in the life sciences. Progress has relied on scientific acumen and on technological advances that provide detailed insights into processes at the atomic, molecular, multisubunit complex, cellular and sometimes organismal levels. These many successes, however, should not blind us as to how our knowledge is incomplete and error-prone. Virtually all (99.85%) protein sequences have no associated experimental evidence at the protein level and for 52% their annotations are flagged as containing possible errors (www.ebi.ac.uk/uniprot/TrEMBLstats). Furthermore, scientific knowledge from targeted studies has been gained unevenly: of all human brain-expressed genes for example, science has focused on very few, with the top 5% of such genes being the subject of 70% of the literature [1].

Whole-genome experiments seek to address these deficiencies of uneven coverage and incompleteness. These are aided by technological innovations that inexorably generate ever larger data sets. Critically, however, big data analysis *per se* reveals not mechanistic causes, but rather correlations and patterns, and leaves questions starting *Why* unanswered [2]. Even when subsequent experiments address more narrowly defined hypotheses while exploiting this data, these also often fail to determine causality. Correlations and patterns may describe the data set well, but they need to be supplemented by causal inferences in order to predict phenomena reliably. The transformation of large, unstructured data sets to insights (Figure 1) and predictive biology is challenging and rarely attained.

**Figure 1.**
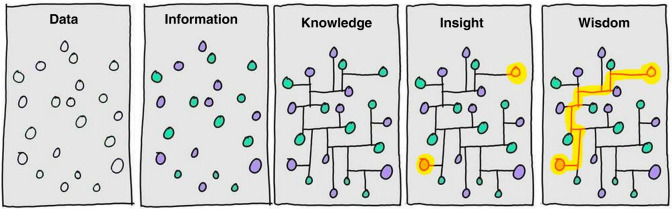
Information isn't. ‘Data is not information, information is not knowledge, knowledge is not understanding, understanding is not wisdom’ (Clifford Stoll and Gary Schubert). The drawing nicely captures some of the distinguishing features of these concepts. Wisdom should also permit reliable prediction. Illustration by David Somerville from original drawings by Hugh McLeod, reproduced with permission (personal communication).

In human genomics, data and annotations have grown rapidly. The 3.2 billion base reference genome is partitioned currently into 20 338 protein-coding and 22 521 non-protein-coding gene annotations that are transcribed into 200 310 transcripts (www.ensembl.org/Homo_sapiens/Info/Annotation) that start from 308 214 locations [3]. Binding sites, often considered to regulate the activity of these genes, have been assigned to 636 336 regions occupying 8.1% of the genome [4]. Nevertheless, experiments imply that many protein–DNA and protein–RNA binding events are not consequential (i.e. are not functional) [5,6]: molecular events are often ‘noise’ that have no subsequent bearing on whether a cell or organism thrives and propagates [7].

Human genome annotation is an incomplete, undoubtedly biased and error-prone molecular parts list. Long-read [8] or targeted RNA sequencing [9] often reveals new or erroneous transcript models, even in well-annotated loci, and predictions of enhancers produce largely discordant results [10]. The value of annotations generated by large-scale big data ‘omics projects’, such as ENCODE [4], FANTOM5 [3] and the Human Cell Atlas [11], is not an immediate gain of new biological knowledge. Instead, their value is technical, by providing new standards, analytical approaches and reagents, as well as data that is accessible, standardised, reusable and extensive that can be exploited by anyone in order to frame new mechanistic hypotheses.

Despite these issues, genomics is not destined forever to produce only large annotation data sets of uneven completeness, quality and predictive potential. Rather the introduction of three novel approaches, based on emerging technologies and analytical methods, could transform its ability to make causal inferences and to address hypotheses. Critically, each is founded on DNA changes that causally lead to downstream effects; reverse causation — DNA mutation caused by phenotypic change — is excluded.

The first of these combines two recent technologies, namely single-cell genomics and multiplexed genome sequence editing by CRISPR/Cas9 [12–15], to couple individual genomic perturbations to transcriptomic read-outs in each of many single cells. Applications have investigated the downstream cellular effects of knocking out transcription factors [14] or genes involved in the unfolded protein [12] or the immune response [15]. When applied across the human genome, there is potential to determine how each DNA lesion causally alters a cell's survival, differentiation or proliferation thereby aiding the generation of more targeted functional hypotheses. Beyond the manipulation of single human cells and the editing of their genomes, it is hoped that real-time image-based high-content screening [16], real-time sampling of a living cell's contents [17] and spatial transcriptomics [18] will together provide the infrastructure required to generate and test functional hypotheses at the genome scale.

The second innovation also links DNA variation to phenotype, but at the human population not cellular level. Sequencing exomes of large cohorts, over half-a-million strong, is predicted to identify at least 7.5% of all possible loss-of-function variants, defined as point substitutions that either introduce stop codons or disrupt splice sites in protein-coding genes [19]. These variants are naturally occurring alleles whose deleterious effects result in their preferential loss from the population and cause their population frequencies to be lower than otherwise expected. Population-scale genome sequencing [20] thus will reveal an increasing number of functional sites whose mutation reduces reproductive success.

The final innovation is Mendelian randomisation. This approach applies the framework of randomised controlled trials to DNA variants that have a robust correlation with a modifiable exposure or biological intermediate [21,22]. In a first step, DNA variants are identified that predict the life-long levels of, and thus exposure to, a molecule. In the next step, these variants are tested for the extent by which they explain a complex trait or disease risk. For example, four DNA variants were found that showed genome-wide significance in their prediction of 25-hydroxyvitamin D (25OHD) levels; then, it was calculated that a two-fold increase in multiple sclerosis disease risk is conferred by a combination of these alleles that reduces 25OHD levels, in a genetically determined manner, by an amount equal to 1 s.d. in log-transformed values [23]. The applicability of Mendelian randomisation has been substantially broadened by exploiting DNA variants that predict RNA [24,25] and protein [26,27] levels to test for a causal effect on traits or disease risk. While challenges need to be overcome, most specifically that of horizontal pleiotropy [28], Mendelian randomisation has potential to reveal causal relationships between DNA variant and trait, and between trait pairs [29].

As sequence data becomes cheaper and easier to generate, its acquisition will be ever more torrential. Nevertheless, in order to generate knowledge, this data needs first to be structured into reliable annotation before being used with approaches that predict causal relationships. Correlation alone will never be sufficient to determine function over effect or causation over statistical association. In time, our currently patchy knowledge will grow and join up. How big will we need big knowledge to be? Measuring a single phenotype caused by the substitution or deletion of each nucleotide in a human genome in, say, 2000 cell types would result in over 24 trillion observations. Yet, even this experiment would fail to account for cellular variation due to state, development, cancer, epistasis or external stimuli. Clearly, this is a path we are just beginning to tread.

## Summary

- Life sciences are awash with data, but relatively bereft of knowledge.
- Human genome sequence annotations are extensive yet are incomplete, often inconsistent and error-prone, and fail to represent functional knowledge.
- Answering *Why* questions requires a detailed understanding of cause-and-effect, rather than correlations and statistical associations.
- Coupling single-cell transcriptomics to CRISPR/Cas9 genome editing, population-scale genome sequencing and Mendelian randomisation each has the potential to identify functions without being confounded by reverse causation.
- Genomic data is easy and relatively cheap to generate. The critical question is not whether such data can be generated, but whether it ought to be: specifically, whether it will generate new knowledge and have a high predictive value.

## Abbreviations

25OHD: 25-hydroxyvitamin D
